# Magnetic Elastomers with Smart Variable Elasticity Mimetic to Sea Cucumber

**DOI:** 10.3390/biomimetics4040068

**Published:** 2019-10-09

**Authors:** Yusuke Kobayashi, Shota Akama, Suguru Ohori, Mika Kawai, Tetsu Mitsumata

**Affiliations:** 1 Graduate School of Science and Technology, Niigata University, Niigata 950-2181, Japan; f19b131j@mail.cc.niigata-u.ac.jp (Y.K.); aaa.rock@icloud.com (S.A.); mikagoro@eng.niigata-u.ac.jp (M.K.); 2 ALCA, Japan Science and Technology Agency, Tokyo 102-0076, Japan; 3 Graduate School of Engineering and Science, Yamagata University, Yonezawa 992-8510, Japan; suguru.izumo@gmail.com

**Keywords:** stimuli-responsive material, magnetic elastomer, magnetic gel, viscoelastic property, magnetorheology, biomimetic, sea cucumber

## Abstract

A magnetic-responsive elastomer consisting of magnetic elastomer and zinc oxide with a tetrapod shape and long arms was fabricated mimetic to the tissue of sea cucumber in which collagen fibrils are dispersed. Only the part of magnetic elastomer is active to magnetic fields, zinc oxide plays a role of reinforcement for the chain structure of magnetic particles formed under magnetic fields. The magnetic response of storage modulus for bimodal magnetic elastomers was measured when the magnetic particle was substituted to a nonmagnetic one, while keeping the total volume fraction of both particles. The change in storage modulus obeyed basically a mixing rule. However, a remarkable enhancement was observed at around the substitution ratio of 0.20. In addition, the bimodal magnetic elastomers with tetrapods exhibited apparent change in storage modulus even at regions with a high substitution ratio where monomodal magnetic elastomers consist of only magnetic particles with less response to the magnetic field. This strongly indicates that discontinuous chains of small amounts of magnetic particles were bridged by the nonmagnetic tetrapods. On the contrary, the change in storage modulus for bimodal magnetic elastomers with zinc oxide with irregular shape showed a mixing rule with a substitution ratio below 0.30. However, it decreased significantly at the substitution ratio above it. The structures of bimodal magnetic elastomers with tetrapods and the tissue of sea cucumber with collagen fibrils are discussed.

## 1. Introduction

Stimuli-responsive polymer gels mimetic to biological systems have been widely investigated during the last few decades. Normally, gel is swollen by a large amount of solvent such as water. Therefore, gel is very soft compared to unswollen materials such as plastics, woods, or metals. In addition, gel is an open-system material so that chemical substances in the gel can be transported and communicated with those outside. These characteristics, in their physical properties are similar to the biological tissues. That would be why gels have been widely used as a biomimetic material that undergoes drastic changes in physical properties by physical stimuli. Many achievements of biomimetic polymer gels have been reported in, for example, these reviews [1,2,3].

**Figure 1:**
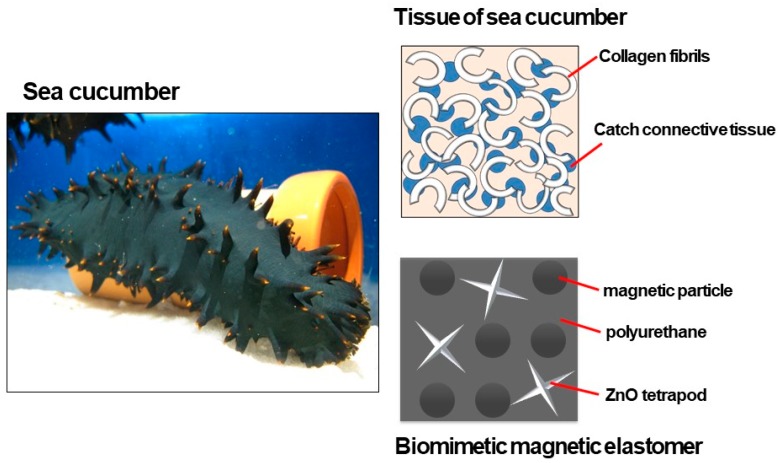
Photograph of a sea cucumber and schematic illustration representing the tissue of sea cucumbers, consisting of catch connective tissue and collagen.

It is well known that the skin of sea cucumber demonstrates drastic changes in elastic modulus by a physical stimulus. Sea cucumbers are categorized as *echinoderms*, as well as sea urchins and star fish. A schematic illustration of the tissue of sea cucumber is shown in Figure 1. Tissues called catch connective tissue, where the elasticity changes by a physical stimulus, when it is in the vicinity of collagen fibrils which are dispersed in a soft tissue of sea cucumber [4,5,6,7,8,9,10,11]. Many pieces of collagen with high elasticity are also dispersed in the soft tissue. When a stimulus is applied to the sea cucumber, the catch connective tissue changes to a hard state resulting in elasticity increasing over the whole of the body. The pieces of collagen play a role as reinforcing materials, similarly to fillers in polymer composites.

Some stimuli-responsive materials mimetic to sea cucumber have been developed [12,13,14,15,16,17]. For example, Capadpna et al. synthesized a rubbery host polymer and rigid cellulose nanofiber and found that the material exhibited a reversible reduction by a factor of 40 of the tensile modulus (from 800 to 20 MPa), due to nanofibers interacting with temperature changes [12]. Qian et al. synthesized a hydrogel consisting of bacterial cellulose and polyelectrolyte and they found that the compression modulus for the gel changed from 3.8 kPa to 1.1 kPa by ionic strength changes [13]. Gao et al. synthesized a supramolecular polymer hydrogel doped with Ca^2+^ ions and found that the mechanical properties changed responding to pH and Ca^2+^ concentration changes [14].

We have investigated so far, magnetic-field-responsive gels and elastomers, that exhibit drastic changes in elastic modulus by an application of magnetic fields. A magnetic gel or elastomer with magnetic particles exhibited a significant increase in storage modulus exceeding 500 times with respect to the off-field modulus [18,19]. Moreover, bimodal magnetic elastomers consisting of both magnetic and nonmagnetic particles demonstrated an enhanced magnetic response due to the chains of magnetic particles associated with nonmagnetic ones [20,21,22,23,24,25,26]. These results indicate that the nonmagnetic particle functions as an intermediator of stress transfer between the chains of magnetic particles although the particles are not moved by magnetic fields. This mechanism is similar to that for the modulus change of sea cucumbers. The catch connective tissue and collagen fibrils in sea cucumbers correspond to magnetic elastomer and nonmagnetic particles in magnetic elastomers, respectively.

In the previous studies [20,21,22,23,25], nonmagnetic particles with irregular shape were employed to reinforce the chain structure of magnetic particles. In this study, we used nonmagnetic particles with a tetrapod shape and investigated the effect of particle shape on the magnetorheological effect (MR effect) for bimodal magnetic elastomers. It is because biologists consider the elasticity change of sea cucumbers to vary depending on the shape of collagen fibrils; e.g., no hardening occurs for collagens of the anchor of ship in *Synapta maculata* or the wheels of vehicle in *Polycheira rufescens*. The tetrapod used in this study was a single crystal of zinc oxide (ZnO), which is the same chemical composition as ZnO with irregular shape, which was also used as a comparison. The effect of particle shape of ZnO insensitive to magnetic fields and the role of collagen fibrils in sea cucumbers are discussed.

## 2. Materials and Methods

### 2.1. Synthesis of Bimodal Magnetic Elastomer

**Figure 2:**
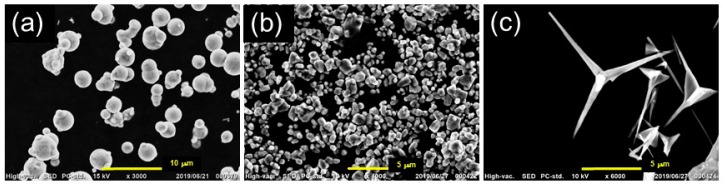
Scanning electron microscope (SEM) photographs for (**a**) magnetic particles, (**b**) nonmagnetic particles (irregular shape), and (**c**) nonmagnetic tetrapods.

Polypropylene glycols (P2000, G3000B, Adeka Co., Tokyo, Japan) with molecular weights of *M*_w_ = 2000 and 3000 were used for the matrix of magnetic elastomers. Tolyrene diisocyanate (Wako Pure Chemical Industries. Ltd., Osaka, Japan) and dioctyl phthalate (DOP, Wako Pure Chemical Industries. Ltd., Osaka, Japan) were used for a crosslinker and plasticizer, respectively. Carbonyl iron (CI, CS Grade BASF SE., Ludwigshafen am Rhein, Germany) was used for magnetic particles. Zinc oxide, ZnO, with an irregular shape (LPZINC-2 Sakai industries Co., Japan) and a tetrapod shape (WZ-0511, Panasonic, Japan) were used as nonmagnetic fillers. The scanning electron microscope (SEM) photographs for these particles are presented in Figure 2. The median diameter of carbonyl iron and ZnO particles was 7.0 ± 0.2 and 2.1 ± 0.2 μm, respectively, determined by a particle size analyzer (SALD-2200, Shimadzu Co. Ltd., Kyoto, Japan). The length and width of the arm for tetrapod ZnO was 8 and 0.4 μm, respectively, determined by SEM photographs. The saturation magnetization of carbonyl iron particles was measured to be 190 emu/g by SQUID magnetometer (MPMS, Quantum Design Inc., San Diego, CA, USA).

Magnetic elastomers were synthesized using a prepolymer method. The molar ratio of -NCO to -OH group for the prepolymer was constant at 2.01 (=[NCO]/[OH]). All particles were mixed with prepolymer, linear polymer, plasticizer, and catalyst. Ultrasonication was carried out for the liquid. The mixed liquid was poured into a silicon mold and cured for 30 min at 100 °C in a vacuum oven. The weight concentration of DOP to the matrix without magnetic particles was fixed at 40 wt%. The volumic substitution ratio of nonmagnetic particles was calculated from *φ*_ZnO_/*φ*_total_ (*φ*_ZnO_: volume fraction of ZnO, *φ*_total_ = *φ*_ZnO_ + *φ*_CI_) and it varied from 0 to 1, while keeping the total volume fraction of magnetic and nonmagnetic particles at *φ*_total_ = 0.24. The density of magnetic elastomers obtained was measured by a densimeter (MD-300S, Alfa Mirage, Japan) to determine the actual volume fractions of magnetic and nonmagnetic particles. The density of magnetic particles, ZnO particle, ZnO tetrapod and polyurethane was determined to be 7.565, 5.813, 5.806, and 1.0 g/cm^3^, respectively.

### 2.2. Rheological Measurements

Magnetic response of the storage modulus at 1 Hz, at a strain of *γ* = 10^−4^ for magnetic elastomers, was measured by dynamic viscoelastic measurements using a rheometer (MCR301, Anton Paar Pty. Ltd., Graz, Austria) with a nonmagnetic parallel plate (PP20/MRD). The measurement was carried out at 20 °C. The sample was a disk 20 mm in diameter and 1.0 mm thick. The magnetic field strength was 500 mT, and the direction of the magnetic field was parallel to the sample thickness. Magnetization curves for CI particles were reported in our previous study [27]. The storage modulus data was determined by averaging over three different samples in a single batch.

### 2.3. SEM Observations

The shape of magnetic and nonmagnetic particles in the powder state and the particle morphology for magnetic elastomers were observed using scanning electron microscopy (SEM, JCM-6000 Neoscope JEOL Ltd. Tokyo, Japan) with an accelerating voltage of 10 kV without Au coating.

## 3. Results and Discussion

**Figure 3:**
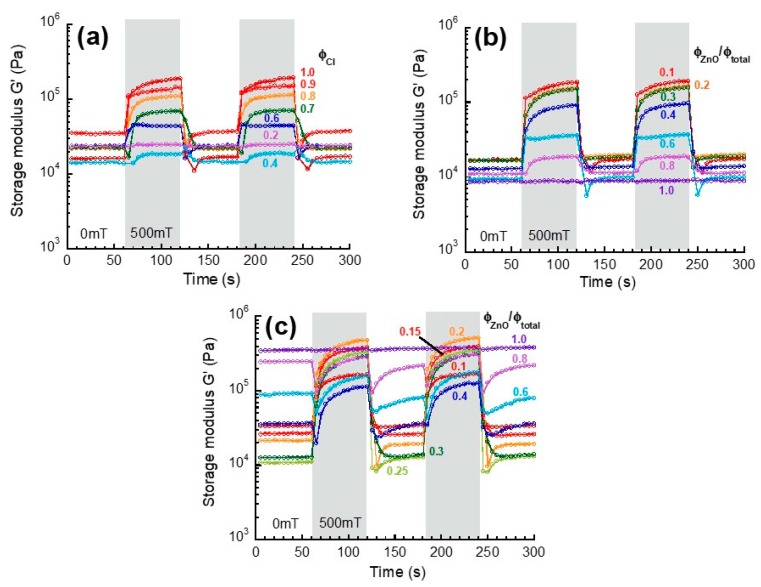
Magnetic-field response of storage modulus in the linear viscoelastic regime for (**a**) monomodal magnetic elastomer and bimodal magnetic elastomers containing (**b**) nonmagnetic particles or (**c**) nonmagnetic tetrapods (Frequency 1 Hz, Strain 10^−4^).

Figure 3a–c show the magnetic-field response of storage modulus at a strain of 10^−4^ (in the linear viscoelastic regime) for monomodal magnetic elastomers, bimodal magnetic elastomers with nonmagnetic particles, and bimodal magnetic elastomers with tetrapods, respectively. Both monomodal and bimodal magnetic elastomers except for *φ*_ZnO_/*φ*_total_ = 1.0 demonstrated a pulsatile response synchronized with the application of the magnetic field. It is natural that the magnetic response for bimodal magnetic elastomers showed a trend to diminish by substituting magnetic particles to nonmagnetic particles (i.e., increasing the substitution ratio). No magnetic response in the storage modulus was observed for bimodal magnetic elastomers with only nonmagnetic particles or tetrapods, although a bulk of ZnO exhibited weak diamagnetic character [28].

**Figure 4:**
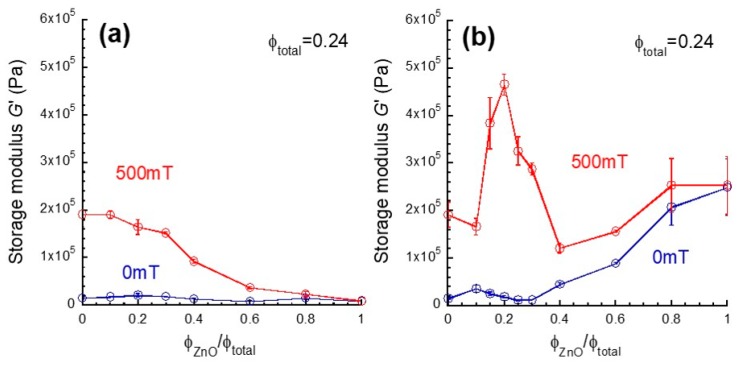
Storage modulus at 0 and 500 mT for bimodal magnetic elastomers containing nonmagnetic (**a**) particles or (**b**) tetrapods as a function of the substitution ratio (Frequency 1 Hz, Strain 10^−4^).

Figure 4a presents the magnetic field effect on the storage modulus at a strain of 10^−4^ for bimodal magnetic elastomers with nonmagnetic particles as a function of the substitution ratio. The storage modulus at 0 mT was nearly independent of the substitution ratio, suggesting the percolation did not occurred by the substitution. At 500 mT, the storage modulus kept high values of storage modulus at *φ*_ZnO_/*φ*_total_ < 0.2. This indicates that the stress transfer among magnetic particles occurs via nonmagnetic particles, resulting in the formation of continuous chains of both magnetic and nonmagnetic particles. At *φ*_ZnO_/*φ*_total_ > 0.3, the storage modulus at 500 mT largely decreased with the substitution ratio, indicating that the number density of composite chains of magnetic and nonmagnetic particles decreased with the substitution ratio. Figure 4b shows the magnetic field effect on the storage modulus for bimodal magnetic elastomers with nonmagnetic tetrapods as a function of the substitution ratio. The storage modulus at 0 mT was constant at *φ*_ZnO_/*φ*_total_ < 0.3, indicating significant agglomerates or particle network of nonmagnetic tetrapods was not formed in the magnetic elastomer at this region. Meanwhile it gradually increased with the substitution ratio at *φ*_ZnO_/*φ*_total_ > 0.3, suggesting that nonmagnetic tetrapod developed to a particle network in the polyurethane matrix. At 500 mT, no change in the storage modulus was observed at *φ*_ZnO_/*φ*_total_ < 0.1, showing that the few chains associated with nonmagnetic tetrapods are not percolated. Interestingly, the storage modulus demonstrated a significant peak at around *φ*_ZnO_/*φ*_total_ = 0.2 with two onset ratios of 0.1 and 0.4. It is considered that the lower onset is caused by a percolation for nonmagnetic tetrapods and the higher onset is due to the percolation for magnetic particles. At *φ*_ZnO_/*φ*_total_ > 0.4, the storage modulus at 500 mT increased with the substitution ratio similar to the behavior of storage modulus at 0 mT.

**Figure 5:**
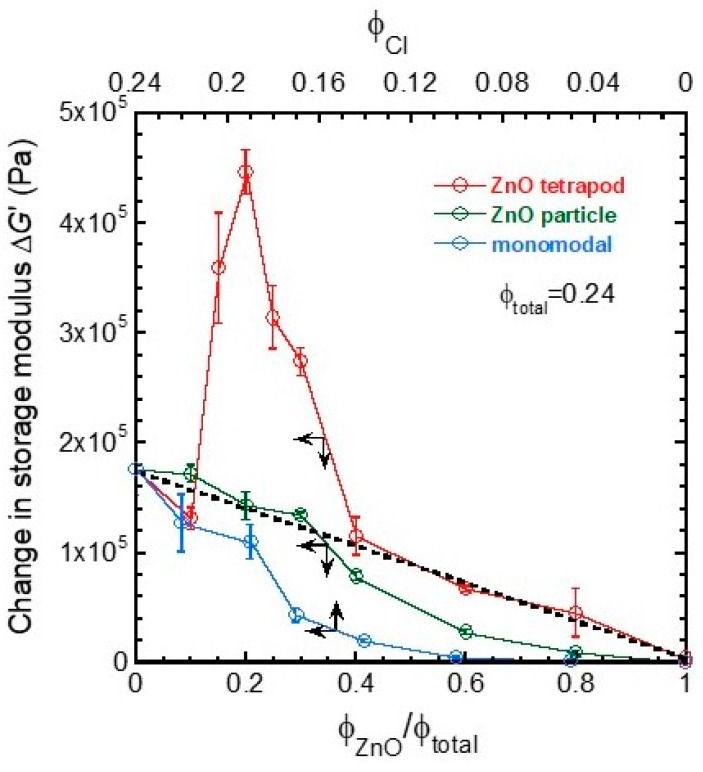
Change in storage modulus for bimodal magnetic elastomers containing nonmagnetic particles or tetrapods as a function of the substitution ratio. The change in storage modulus for monomodal magnetic elastomers with the same volume fraction of magnetic particles as bimodal magnetic elastomers, is also shown (Frequency 1 Hz, Strain 10^−4^).

**Figure 6:**
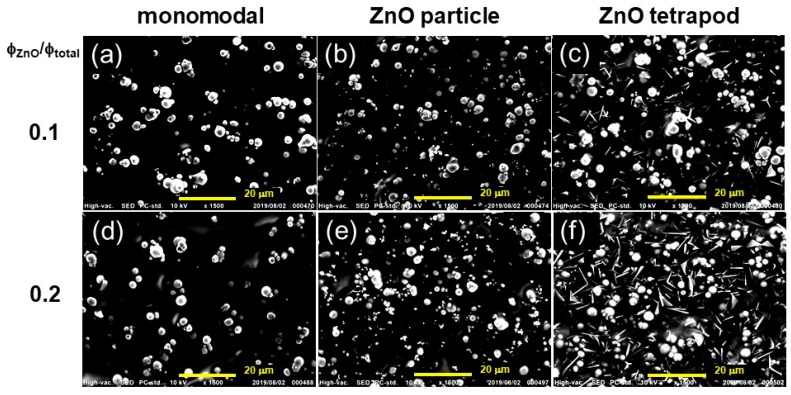

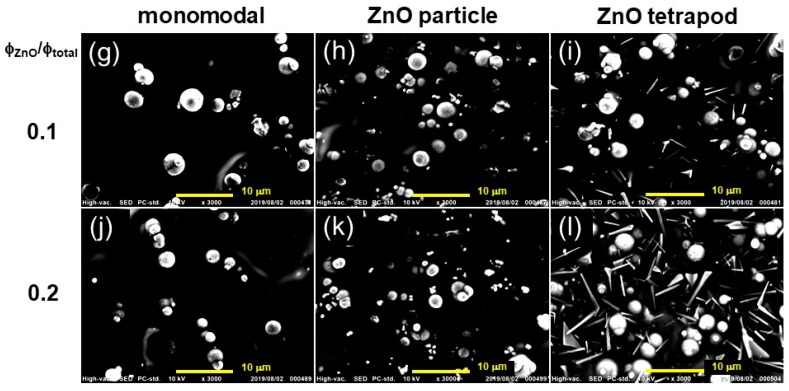
SEM photographs for (**a**,**d**,**g**,**j**) monomodal magnetic elastomers, (**b**,**e**,**h**,**k**) bimodal magnetic elastomers with nonmagnetic particles and (**c**,**f**,**i**,**l**) bimodal magnetic elastomers with nonmagnetic tetrapods at substitution ratios of *φ*_ZnO_/*φ*_total_ = 0.1 and 0.2. Magnification (**a**–**f**) × 1500 and (**g**–**l**) × 3000.

Figure 5 shows the change in storage modulus for bimodal magnetic elastomers with nonmagnetic particles and tetrapods as a function of the substitution ratio. The relation between the storage modulus and the volume fraction of magnetic particles for monomodal magnetic elastomers is also presented in the same figure; the top *x*-axis represents the volume fraction of magnetic particles. The volume fraction of magnetic particles for monomodal magnetic elastomers is the same as those for bimodal magnetic elastomers. The change in storage modulus Δ*G* was calculated from Δ*G* = *G*_500_ − *G*_0_, where *G*_0_ and *G*_500_ are the storage modulus at 0 and 500 mT, respectively. The Δ*G* for monomodal magnetic elastomers was 1.76 × 10^5^ Pa at *φ*_CI_ = 0.24 and it monotonously decreased with the volume fraction of magnetic particles, which is a typical dependency seen in magnetic elastomers. The increase in Δ*G* was observed at *φ*_CI_ = 0.10 where is the percolation threshold for monomodal magnetic elastomers containing only carbonyl iron particles with a diameter of 7.0 μm. This value is slightly higher than that for carbonyl iron particles with a diameter of 2.5 μm (*φ*_CI_ = 0.04) [29]. The Δ*G* for bimodal magnetic elastomers with nonmagnetic particles obeyed a mixing rule only at *φ*_ZnO_/*φ*_total_ < 0.3; Δ*G* = (*φ*_ZnO_/*φ*_total_)Δ*G*_CI_ + (1 − *φ*_ZnO_/*φ*_total_)Δ*G*_ZnO_, where Δ*G*_CI_ = 1.76 × 10^5^ Pa and Δ*G*_ZnO_ = 0 Pa. This result coincides with the previous results that the change in storage modulus was enhanced by nonmagnetic particles with irregular shape at similar substitution ratio [20]. At *φ*_ZnO_/*φ*_total_ > 0.4, the Δ*G* decreased with the substitution ratio showing under-deviation from the mixing rule. On the other hand, the Δ*G* for bimodal magnetic elastomers with tetrapods decreased with the substitution ratio at *φ*_ZnO_/*φ*_total_ < 0.1 as well as monomodal magnetic elastomers, meaning that the chain structure of magnetic particles is affected by neither a decrease in the number of magnetic particles nor an addition of nonmagnetic tetrapods. As described in Figure 4, we observed an interesting phenomenon, where the Δ*G* had a maximum of 4.46 × 10^5^ Pa at *φ*_ZnO_/*φ*_total_ = 0.2, which is higher than twice of that for monomodal magnetic elastomers. There was no magnetic interaction between ZnO tetrapods/particles because they have no magnetic moment themselves. On the other hand, there is a certain magnetic interaction between CI particles, resulting in the chain formation of CI particles under the magnetic field. As seen in Figure 5, the change in storage modulus for monomodal magnetic elastomer with only CI particles was much lower than that for ZnO tetrapods (~2/9). Therefore, the enhanced magnetorheology seen at *φ*_ZnO_/*φ*_total_ = 0.2 was caused by only mechanical interaction between ZnO tetrapod and the chain of CI particles. The simple mixing rule suggests the parallel connection of modulus changes for magnetically active domains and nonmagnetic ones. This means that magnetically active domains are homogeneously distributed in the bimodal elastomer. Tamori et al. described that the mechanical behavior of the holothurian dermis shows it can be regarded as a fiber-reinforced composite material in which fiber with a high elastic modulus are embedded in hydrogels with a low elastic modulus [7]. It would be of interest to see the statistical analysis for the percolation threshold of collagen fillers in sea cucumber. Interestingly, the Δ*G* for bimodal magnetic elastomers with tetrapod at *φ*_ZnO_/*φ*_total_ > 0.4 decreased with the substitution ratio satisfying the mixing rule. Surprisingly, the Δ*G* for bimodal magnetic elastomers with tetrapods was far higher than those for nonmagnetic particles. This result strongly indicates that only nonmagnetic tetrapods connect between discontinuous chains of magnetic particles even though the chain is extremely short. At *φ*_ZnO_/*φ*_total_ = 0.6, the Δ*G* for magnetic elastomers with only magnetic particles was far lower than that of nonmagnetic tetrapods (~1/14), meaning that the discontinuous chains of magnetic particles are connected via nonmagnetic tetrapods resulting in the percolation of the chains through the sample. Many ZnO rods unintentionally produced by breaking tetrapods were observed for SEM photographs (particularly in Figure 6l). We considered the enhanced magnetorheological effect to need at least a ZnO rod with 2D structure and a high aspect ratio, although ZnO tetrapods with 3D structure would be much more effective for mechanical-stress transfer.

Figure 6 displays the SEM photographs for monomodal magnetic elastomers, bimodal magnetic elastomers with nonmagnetic particles, and bimodal magnetic elastomers with tetrapods at substitution ratios of *φ*_ZnO_/*φ*_total_ = 0.1 and 0.2. As seen in Figure 6a, monomodal magnetic elastomers exhibited some secondary particles in the polyurethane matrix, which coincides with our previous report investigating the effect of sonication on the dispersibility of magnetic particles [30]. Figure 6b,c demonstrate that both nonmagnetic particles and tetrapods are randomly dispersed in a polyurethane matrix without forming secondary particles. It can be seen from Figure 6e,f that nonmagnetic tetrapods are densely embedded in the matrix compared to the nonmagnetic particles although the substitution ratio where the *φ*_ZnO_/*φ*_total_ was completely the same. A similar trend in particle morphology was also observed for the enlarged photographs in Figure 6k,l. We reported a result of in-situ observation by computed tomography that magnetic particles form a chain structure within polyurethane elastomers by applying a magnetic field [31]. It is well known that sea cucumbers have hard collagen tissues in their soft dermis to protect themselves from enemies. Some sea cucumbers have only bundles of collagen fibrils, but others have both collagen fibrils and polygon-shaped collagen. For example, Thurmond et al. reported that the tissue of *Cucumaria frondosa* has spaces between bundles of collagen fibrils which are occupied principally by microfibrils [11]. Tamori et al. showed SEM photographs for the tissue of *Holothuria leucospilota* and *Stichopus chloronotus* in both soft and hard states [7]. The photographs indicate that collagen fibers exist in dermis of the sea cucumbers at a high volume fraction. Moreover, they proposed striking evidences that the collagen fibers are connected via catch connective tissues (cross-bridges) and the number of cross-bridges connecting collagen fibers was increased in the stiff state. They also reported a novel protein in the dermis, softenin, for decreasing the dermal stiffness through inhibiting cross-bridge formation between collagen fibrils [32].

## 4. Conclusions

Magnetic-field responsive elastomers mimetic to the composite structure of sea cucumbers, which catch connective tissue and collagen fibrils were fabricated, and the shape effect of the nonmagnetic particles on the change in elasticity was investigated. It was observed that for bimodal magnetic elastomers with tetrapods, that the magnetic response in storage modulus was dramatically enhanced at a substitution ratio of 0.2. The enhanced effect was caused by the percolation of stimuli-responsive parts consisting of discontinuous chains of magnetic particles and nonmagnetic tetrapods. This result proposes a design for a smart materials, in which the constituent of stimuli-responsive material should only be ~20 vol% of the bulk material, i.e., 80 vol% of the bulk does not react to the stimulus. We imagine that most of biology has this kind of structure or a similar mixing ratio in order to generate significant and effective stimuli-responsive properties with small amounts of energy. It would be of interest to analyze the mixing ratio of stimuli-responsive tissues in biological systems using the percolation theory. We anticipate that the findings obtained in this study can be applied to designing new magneto-active soft materials, demonstrating drastic changes in viscoelastic property by using weak magnetic fields.
